# Barriers to health care access in a federally under-resourced system: experiences of Alzheimer’s disease and related dementias patients and caregivers in Puerto Rico

**DOI:** 10.3389/fpubh.2026.1923342

**Published:** 2026-10-08

**Authors:** Mariela Torres-Cintrón, Alexandra Mendoza-Graf, Gabriela Castro, Gilberto Ramos-Valencia, Marielena Lara, Ivonne Z. Jiménez-Velázquez, Ilka Rios, Johan Hernandez-Santiago, Beverly Weidmer, Justin W. Timbie

**Affiliations:** 1 University of Puerto Rico, Medical Sciences Campus, San Juan, Puerto Rico; 2 RAND, Santa Monica, CA, United States; 3 RAND, Arlington, VA, United States

**Keywords:** Alzheimer’s disease, caregivers, dementia, health care access, health disparities, long-term services and supports, Medicare Advantage, Puerto Rico

## Abstract

**Background:**

Alzheimer’s disease and related dementias have reached crisis proportions in Puerto Rico, where the proportion of residents aged 65 and older has nearly doubled in the past decade, from 11 to 21%, making it one of the oldest populations in the Americas. Despite near-universal insurance coverage, families frequently struggle to translate potential access into actual care utilization, yet the systemic barriers driving this gap remain poorly documented.

**Methods:**

This qualitative study examined barriers to dementia care access among individuals with Alzheimer’s disease and related dementias and their caregivers across Puerto Rico. Semi-structured interviews were conducted with 51 patient–caregiver dyads representing 19 municipalities between December 2023 and November 2024. Data was analyzed using a directed content analysis approach guided by Andersen’s Behavioral Model of Health Services Use, with barriers categorized across structural, process-level, and enabling resource domains.

**Results:**

Extended waiting times, limited specialist access, and inadequate care coordination affected nearly all families. Structural underinvestment, particularly the absence of Medicaid long-term services and supports and restrictive Medicare Advantage networks, further constrained potential access. Despite 94% of participants being enrolled in Medicare Advantage, insurance coverage did not translate into realized access to needed services.

**Conclusion:**

The universality of access barriers reflects systemic rather than individual-level failures in dementia care in Puerto Rico. Health systems serving older adults with dementia should prioritize open-access scheduling, integrated care coordination, and routine screening for social needs. Federal policy reforms, including Medicaid long-term services and supports parity for United States territories and expansion of dementia-specific care models, are essential to achieving equitable access for this population.

## Introduction

Alzheimer’s disease and related dementias (AD/ADRD) have reached crisis proportions in Puerto Rico. Driven by rapid population aging, the proportion of residents aged 65 and older has nearly doubled in the past decade, from 11 to 21%, making Puerto Rico one of the oldest populations in the Americas (1). As a result, approximately one in eight adults aged 65 or older currently lives with dementia, and Alzheimer’s disease now ranks as the fourth leading cause of death on the island (2–5). Furthermore, the number of affected individuals is projected to double through 2050, further stressing the territory’s capacity to deliver accessible, high-quality care for this population (6). At the same time, nearly half of all dementia cases remain undiagnosed, leaving many families to manage cognitive, behavioral, and functional decline without guidance or support (4).

Long-standing structural and policy inequities in federal financing and local infrastructure severely constrain the territory’s ability to respond to this growing demand for care. Puerto Rico receives capped Medicaid funding through a block-grant system rather than open-ended matching as in the U.S. mainland, resulting in chronic underfunding. Medicare Advantage payment rates are likewise 43 percent lower than in the lowest-paid U.S. states, constraining provider participation and limiting access to specialists (7, 8). These fiscal disparities coincide with the absence of Medicaid coverage for long-term services and supports (LTSS), leaving families without publicly funded options for nursing-home care, adult day care, or in-home personal care (9).

Geography, natural disasters, demographic shifts, and decades of slow economic growth have converged to further compound Puerto Rico’s health care challenges. Healthcare facilities and specialists are heavily concentrated in the San Juan metropolitan area, requiring residents of mountainous and rural municipalities to travel long distances for specialty services. The devastation caused by Hurricane María and subsequent natural disasters damaged health infrastructure and triggered extensive physician migration (10), while the ongoing outmigration of younger adults has increased the proportion of older adults and depleted both the formal health care workforce and informal caregiving supports (1, 10). Taken together, these interlocking pressures have produced a health care system that is increasingly fragile and fragmented, in which older adults encounter multiple barriers to accessing essential care. For people living with AD/ADRD, these weaknesses are magnified: optimal dementia care depends on coordinated specialty services, LTSS, and comprehensive care management, services particularly vulnerable to underinvestment (11).

Despite an expanding literature on dementia care access, most U.S.-based qualitative work has examined care experiences within mainland Medicaid and traditional Medicare systems, offering limited insight into a fully capitated, block-granted, and Medicare Advantage–dominated U.S. territory context that is structurally distinct from the mainland safety net (9). Similarly, while barriers to dementia care have been described broadly in prior work, few studies have explicitly distinguished, using an established access framework, between the structural conditions that limit potential access and the organizational, process-level conditions that limit realized access, a distinction with direct implications for where policy intervention is likely to be most effective. This study addresses these gaps by applying Andersen’s Behavioral Model to systematically categorize and interpret barriers reported by a large, geographically diverse qualitative sample of patient–caregiver dyads within a near-universally insured, Medicare Advantage–dominated population, and by explicitly linking each barrier category to actionable, policy-mutable levers for reform.

This study aimed to identify barriers to dementia care access among individuals living with AD/ADRD and their caregivers in Puerto Rico. Through interviews with 51 patient–caregiver dyads across 19 municipalities, we identified structural factors constraining service availability and process-level barriers impeding use of existing services. Guided by Andersen’s Behavioral Model, which classifies determinants of access as contextual or individual-level, and as predisposing, enabling, or need-related (see Methods) (12). The analysis showed how systematic inequities shape families’ care experiences and offers evidence for policy reform.

## Methods

### Study design

This qualitative study forms part of a larger mixed-methods investigation of dementia care in Puerto Rico (13). It used semi-structured interviews to examine the health care experiences and unmet needs of individuals living with AD/ADRD and their family caregivers in Puerto Rico from Hurricane Maria (September 2017) through 2024. Family members living with AD/ADRD participated directly when their cognitive status permitted.

### Eligibility criteria

Eligibility was assessed at the patient–caregiver dyad level, and both members of each dyad were invited to participate. Individuals with AD/ADRD were eligible if they had a diagnosis of Alzheimer’s disease or a related dementia confirmed by a health care provider, were enrolled in Medicare Advantage or Original Medicare, lived in Puerto Rico at the time of Hurricane María (September 2017), and had an identified family caregiver most knowledgeable about their medical care. Hurricane María was selected as the eligibility anchor, rather than as a pre/post-hurricane comparison point, because it represents a shared, dateable disruption to the island’s health care infrastructure; requiring residence in Puerto Rico at that time ensured that all participating dyads could speak from firsthand experience to the cumulative, longer-term consequences of provider migration, infrastructure damage, and service disruption on their care trajectory over the subsequent 6 years, rather than capturing only recent or isolated experiences.

Capacity to provide informed consent was assessed using a structured teach-back protocol: after each section of the consent form, the interviewer asked the caregiver and patient to restate its content in their own words, revisited any section not clearly understood, and answered questions before proceeding. Written informed consent was obtained from both patients and caregivers, and, when a patient could not demonstrate full consent capacity, from the patient’s caregiver or, where applicable, the patient’s legally authorized representative, consistent with the study’s IRB-approved consent procedures. As appreciation for their participation, caregivers received a $75 VISA gift card, and patients received a small, cognitively engaging gift, regardless of whether the patient’s portion of the interview was completed.

The study protocol was approved by the Institutional Review Board (IRB) of the University of Puerto Rico, Medical Sciences Campus (#2690122/Streamlyne #2311162366), with parallel review by the IRB of RAND (Human Subjects Protection Committee, Protocol #2021-N0674).

### Recruitment and study sample

We recruited a convenience sample of 51 patient–caregiver dyads between November 2023 and November 2024. Caregivers served as the primary informant in each interview; patients were invited to participate directly to the extent permitted by their capacity to consent (see Eligibility Criteria). Of the 51 completed interviews, 24 (47%) were conducted with both the caregiver and the patient present, reflecting the patient’s cognitive capacity to participate directly; 27 (53%) were conducted with the caregiver alone on behalf of a patient with advanced- or moderate-stage AD/ADRD, for whom the caregiver held durable power of attorney or had received the patient’s assent to respond on their behalf.

Recruitment was conducted in partnership with the Alzheimer’s Association of Puerto Rico, the Pro-Alzheimer Care Organization (OPAPA), and the Puerto Rico Primary Care Association, which supports the territory’s 21 Federally Qualified Health Centers (FQHCs). These partners supported outreach by contacting their members directly and distributing a one-page flyer, developed by the research team, that summarized the study’s purpose, eligibility criteria, and participation procedures in Spanish and was shared in both digital and print formats. Members of the research team further expanded outreach by contacting leaders of caregiver support groups and attending community events sponsored by these organizations. Recruitment targeted beneficiaries across the study’s delivery models, with greater representation from FQHCs and vertically integrated medical groups (VIMGs) than from small and large independent medical groups, consistent with the study design. Although formal stratification or quota sampling by demographic characteristics was not employed, recruitment aimed to achieve diversity in geographic region and time since diagnosis. The resulting sample included participants from 19 municipalities across metropolitan and non-metropolitan areas of Puerto Rico (Table 1).

**Table 1:** Demographic and clinical characteristics of patients living with AD/ADRD (*N* = 51).

Characteristic	No.	%
Sociodemographic characteristics		
Female sex	30	59
Mean age, y	—	77
Education beyond secondary school	29	57
Metropolitan area residence	24	47
Nutritional assistance recipient	20	39
Clinical characteristics		
Dementia severity		
Mild	8	16
Moderate	27	53
Severe	16	31
Comorbidities		
Hypertension	30	59
Diabetes	17	33
Cardiovascular disease	15	29
Insurance coverage		
Medicare Advantage	48	94
Dual Medicare-Medicaid eligible	32	63

Between November 2023 and November 2024, 115 caregivers contacted the research team to express interest in participation. Of these, 67 completed eligibility screening, of whom 58 met inclusion criteria; six withdrew due to scheduling conflicts, and one declined participation, resulting in 51 patient–caregiver dyads that completed interviews, reflecting the lag between initial contact, eligibility screening, consent procedures, and scheduling. All 51 complete interviews were included in the analysis.

Rather than applying a predetermined saturation threshold during data collection, recruitment continued throughout the funded study period, and all eligible dyads who completed screening and consented were interviewed. Consistent with the study’s original recruitment plan, which anticipated approximately 50 interviews, the final sample of 51 dyads was achieved through ongoing recruitment rather than a formal saturation assessment (14). This approach reflects an exploratory design intended to capture the full breadth of care experiences across a broad segment of Puerto Rico’s dementia-caregiving population, and the breadth and internal consistency of themes across dyads support the dataset’s adequacy for the study’s exploratory aims.

### Interview procedures

Semi-structured interviews were conducted in Spanish between December 2023 and November 2024, at participants’ homes. Each interview lasted up to 60 min, was audio-recorded, and transcribed verbatim. Trained bilingual interviewers followed a standardized protocol that explored patients’ and caregivers’ experiences with Alzheimer’s care, including perceptions of health, quality of life, and satisfaction with care; access to and coordination of care; attention to social supports and caregiver needs; and the ways in which Hurricane María, Hurricane Fiona, and the COVID-19 pandemic altered health care access and delivery (Table 2). For the portion of the interview involving direct patient participation, interviewers followed established best practices for qualitative research with people living with dementia, including scheduling interviews for times of peak patient alertness, using rapport-building questions, and allowing sufficient response time (15). Interviewers maintained detailed field notes documenting contextual factors and emerging patterns throughout data collection to inform subsequent analysis. All interviews were conducted and transcribed in Spanish, and analysis was performed on the original Spanish-language transcripts to preserve linguistic nuance and cultural meaning. Quotes selected for inclusion in this manuscript were translated into English using AI-assisted translation tools and subsequently verified by a bilingual member of the research team to ensure accuracy and fidelity to the original meaning. The original Spanish-language versions of all translated quotes are provided in Appendix 1 to allow readers to assess potential shifts in tone or emphasis introduced through translation.

**Table 2:** Interview domains of inquiry and sample questions.

Domain of inquiry	Sample question(s)
Patient health status and daily functioning	“Tell me how you feel about your health. Is it excellent, fair, or poor? And why?”—“During the last four weeks, how often have you needed help with your daily activities? Which activities have you needed the most help with?”
Satisfaction with Alzheimer’s care	“Overall, how satisfied are you with the medical care you are currently receiving from your providers for your Alzheimer’s disease? What do you like most about these providers? What do you like least?”
Access to care	“How often are you able to get health care services for your Alzheimer’s disease when you feel you need them?”—“What services do you need that you are not currently receiving?”
Care coordination	“How informed and up to date do your Alzheimer’s care providers seem to be about the care you are receiving from one another?”—“How often do your providers ask you to make a list of all the medications you are currently taking (medication reconciliation)?”
Whole-person care	“How much do you feel your Alzheimer’s care providers listen to and take into account your care priorities?”—“How often do your Alzheimer’s care providers ask you about needs that are not related to medical care—such as your housing situation, utility bills, or access to food?”
Effects of natural disasters on care (Hurricane María, Hurricane Fiona, COVID-19, recent earthquakes)	“How did your care for Alzheimer’s disease change after Hurricane María?”—“How did your care for Alzheimer’s disease change during the COVID-19 pandemic?”
Recommendations for improving care	“How could your medical care be improved, in general or specifically for your Alzheimer’s disease?”—“Is there anything else you would like to add?”

### Data analysis

A directed content analytic approach (16, 17) combined theoretical constructs from the patient-centered medical home model (18) and Andersen’s Behavioral Model (12, 19) with inductive identification of new patterns emerging from the transcripts. Andersen’s Behavioral Model served as the primary organizing structure, guiding the identification of barrier types across structural, process-level, and enabling resource domains and anchoring the distinction between potential and realized access that frames the discussion. The patient-centered medical home model functioned as a complementary lens at the process level, specifically informing codes related to care coordination, continuity, and comprehensiveness of services. Where codes could be mapped onto both frameworks, the research team discussed the primary classification in consensus meetings until agreement was reached. Four experienced qualitative researchers fluent in Spanish conducted coding using Dedoose software guided by a codebook developed by our multidisciplinary team under the leadership of an experienced qualitative methodologist. The coding team jointly reviewed, applied, and iterated on revisions to the codebook. Inter-rater reliability testing produced a Cohen’s *κ* (20) of 0.74 indicating substantial agreement. A total of 1,162 excerpts were coded across the 51 transcripts, exported to Excel, and thematically analyzed, drawing on the team’s Spanish-language fluency to identify linguistic patterns within the original transcripts, to derive overarching themes and variations across subgroups defined by geography, stage of illness, and other characteristics. Andersen’s 1995 revision to the original Aday-Andersen framework (21) introduced three refinements central to this study’s design (12). First, the model became dynamic, incorporating feedback loops in which negative care experiences discourage future care-seeking, a mechanism particularly relevant to a chronic, progressive condition such as AD/ADRD. Second, it distinguished potential access (the availability of services) from realized access (their actual utilization), a distinction that structures the presentation of Results and Discussion throughout this manuscript. Third, it introduced the concept of mutability, prioritizing policy-changeable variables over immutable characteristics and directing analytic attention toward actionable levers for reform. The model has been evaluated extensively through health services research generally (22) and applied specifically within qualitative health services research (23), supporting its suitability for the exploration and theory-informed design used here.

To operationalize Andersen’s Behavioral Model for coding and for the organization of Results, barriers were classified along the two dimensions specified by the framework: level (contextual, i.e., system- or environment-level, versus individual, i.e., household- or person-level) and domain (predisposing, enabling, or need-related factors) (12). Structural Barriers and Disaster-Related Service Disruptions correspond to contextual-level enabling factors, financing structures, provider-network policies, and system-level shocks that determine whether services are available at all, independent of any individual family’s circumstances. Enabling Resource Barriers correspond to individual-level enabling factors and to household- and community-level resources (transportation, income, geographic proximity) that determine whether an eligible family can use available services. Care Process Barriers and Communication and Coordination Failures span both levels, capturing contextual-level organizational and information-exchange practices (e.g., referral systems, scheduling models, EHR interoperability) as they are experienced at the individual level during care navigation. Need-related factors, reflecting the severity and functional impact of AD/ADRD that drives perceived and evaluated need for services, were present throughout all five categories rather than treated as a separate, stand-alone results category, consistent with the uniformly high level of clinical need across this sample (Table 1). This crosswalk between the five results categories and Andersen’s original domains, along with operational definitions and illustrative codes for each, is summarized in Table 3.

**Table 3:** Crosswalk of results categories to Andersen’s behavioral model domains.

Results category	Andersen level	Andersen domain	Operational definition	Illustrative codes
Structural barriers	Contextual	Enabling	System-level financing and policy conditions determine whether dementia-related services exist or are accessible in principle	LTSS coverage gap; MA network restriction
Care process barriers	Contextual → individual	Enabling (organizational)	Administrative, logistical, and organizational practices governing how families navigate and use existing services	Extended waiting time; absent social needs screening
Enabling resource barriers	Individual	Enabling	Household- and community-level resources that determine a family’s capacity to use available services	Transportation barrier; out-of-pocket cost; geographic distance
Communication and coordination failures	Contextual → individual	Enabling (coordination)	Deficiencies in information exchange and referral coordination between providers and families leave caregivers responsible for transmitting medical information	Paper-based referral; lack of EHR integration; repeated clinical history-taking
Disaster-related service disruptions	Contextual	Enabling	Temporary or permanent loss of health care providers and services following natural disasters and public health emergencies, and the resulting gaps in continuity of care	Provider loss after Hurricane María; telehealth’s inability to support clinical examination
(Cross-cutting)	Individual	Need	Severity/functional impact of AD/ADRD shaping perceived and evaluated need for services; embedded within codes across all five categories above, rather than treated as a stand-alone category	Advanced-stage LTSS needs; behavioral symptoms complicating transport

## Results

Patients were predominantly female (59%), with a mean age of 77 years and moderate dementia severity (53%; Table 1). The sample represented 19 municipalities across Puerto Rico, with 53% living in non-metropolitan areas. Educational attainment was relatively high (57% beyond secondary school), yet 39% received food assistance and 63% were dually eligible for Medicare and Medicaid. Most participants (94%) were enrolled in Medicare Advantage plans. Caregivers were predominantly female (82%) and most commonly adult children (53%) or spouses (45%) of the patient.

Guided by Andersen’s (1995) framework, which distinguishes potential from realized access and classifies determinants of access as contextual- or individual-level predisposing, enabling, and need factors (Table 3), our analysis organized barriers into five interrelated categories, three core domains (Structural, Care Process, and Enabling Resource Barriers) and two related extensions (Disaster-Related Service Disruptions and Communication and Coordination Failures), with distinct implications for intervention strategies.

### Structural barriers

Structural barriers are system-level gaps in health care infrastructure and policies that restrict access to essential services and typically require federal-level policy action to address.

#### LTSS coverage gaps

Nearly all the participants described gaps in LTSS coverage as a critical structural barrier. Although some adult day centers operate in Puerto Rico, caregivers noted that these facilities are only available on a self-pay basis and often maintain multi-year waiting lists.

The inadequacy of these services was starkly illustrated by caregivers managing end-stage disease. One caregiver described the overwhelming scope of full-time care needs:

*“She is 24/7. She needs someone. She does not remember anything. She does not feed herself. She does not go to the bathroom by herself. She’s already wearing diapers.”*

Others underscored the profound gap between need and availability:

*“It would be ideal to have these services available, either through your insurance plan or government assistance, but there’s nothing.”*

The nature and urgency of reported barriers varied by disease stage, with caregivers of participants in advanced stages describing immediate LTSS needs:

*“At the stage he’s in now, that’s what he needs. Therapy and physical maintenance help. It’s very important that he delays becoming bedridden.”*

#### Specialist access challenges and provider network issues

Specialist access challenges and provider network issues were widely reported by caregivers, who described difficulty accessing specialists, particularly psychiatrists. Participants also reported that Medicare Advantage network changes often forced patients to change providers despite longstanding care relationships. Rural caregivers described the absence of local specialists, requiring travel to metropolitan areas for care.

As one caregiver explained:

*“Specialists are difficult to access. With the neurologist—goodness—finding a neurologist these days is an uphill battle. There is no special treatment [for people living with Alzheimer’s] for getting an appointment.”*

Closed provider panels frequently excluded new patients altogether:

*“There are neurologists I have called who are not taking new patients. And it has been very difficult. The same with psychiatrists.”*

Network restrictions within MA plans exacerbated these barriers. One caregiver described switching insurers solely to preserve continuity with an established provider:

*“They removed him from the health plan we had and I switched health plans. Just to keep him. Because he treats her very well and has been with her for several years, and honestly, at her age, changing to a new doctor [is difficult].”*

Others reported that plan networks offered little practical access because many specialists declined to contract with MA plans altogether:

*“To get a urologist it has to be one that the plan covers—and most urologists do not take insurance.”*

### Care process barriers

Care process barriers include administrative, logistical, and organizational practices that either burden families with complex procedures or reflect the absence of systems needed to navigate services effectively. These barriers were the most frequently reported access limitations, creating practical obstacles at every step of care-seeking.

#### Extended wait times

Extended wait times dominated this category, with participants describing not only prolonged delays but also their specific effects on individuals with AD/ADRD. One caregiver vividly described the challenge:

*“Appointments with him require waiting four hours—in the waiting room. So yes, for me to take him, I prefer to start early, in the morning.”*

Another caregiver emphasized that appointment times had little practical meaning:

*“For example, I have an appointment at 11 and they schedule four people for 11. At 11:30 still none of the four have been seen.”*

Beyond the time burden, extended waits in crowded facilities provoked agitation and distress among patients:

*“It distresses her too, and many people, because the offices were completely full. I did not want to expose her to that.”*

#### Lack of social needs screening

Lack of social needs screening during medical encounters emerged as a near-universal theme, with most participants reporting that providers rarely assessed housing stability, food security, social isolation, or other factors critical to individuals living with AD/ADRD and their caregivers. The absence of routine screening left many families without referrals to supportive resources available in their communities. When asked how often providers inquired about non-medical needs, caregivers consistently reported that it never happened:

*“I do not think they ever ask, unless they see someone really malnourished.”*

Screening that did occur was reactive rather than systematic:

*“They only communicate with you if they see an urgent need. If there’s no urgency, or you do not raise the concern yourself, they do not ask.”*

One caregiver described having to identify supports independently:

*“They’ve never asked if we needed help. Everything I have and know, I found on my own.”*

### Enabling resource barriers

Enabling resource barriers encompass geographic, financial, transportation, and environmental constraints that limit access to dementia care services. While some factors—such as geography—are immutable, others, including transportation systems and financial assistance, represent policy-mutable areas where targeted reforms could improve equity.

#### Geographic barriers

Geographic barriers reflect Puerto Rico’s concentration of specialists in metropolitan areas. Participants from non-metropolitan municipalities described long travel distances and limited access to specialty care. This uneven distribution of providers created systematic disadvantages for rural residents—an inequitable pattern in which geography, rather than medical need, determined care access. Post-Hurricane María infrastructure damage continues to affect travel routes and provider availability. One family from a rural municipality far from San Juan illustrated the consequences:

*“When she had an episode [of Transient Ischemic Attack] and left the hospital, obviously she could not be alone. I brought both of them [home with me], and I had to change all their doctors to the metropolitan area so I would not have to be running around constantly.”*

Rural caregivers frequently described the complete absence of specialists and services in their municipalities:

*“I searched for places where she could go to a day center. There aren’t any available in the municipality of San Juan. They are available in Carolina, but they do not accept her because of her address.”*

Another remarked:

*“It’s like the services are for the metropolitan area, but here in Humacao it’s completely different.”*

#### Financial barriers

Financial barriers included high transportation costs to reach specialists in distant locations, limited insurance coverage for essential dementia services, and self-pay requirements for adult day centers or respite care. Insurance coverage frequently excluded essential medications, as one caregiver explained:

*“The original medications… the plan does not cover them, and I cannot pay for them. I have to find a way and use the generic ones… For example, the eye drops they need… but they cost $500.”*

Adult day centers and respite care required out-of-pocket payment at prohibitive rates, typically ranging from $900 to $1,200 per month. One caregiver stated:

*“I found another alternative place that’s really cool but super expensive—almost $900 a month. Who can pay that?”*

Financial strain was not limited to current needs; caregivers managing early-stage dementia often anticipated escalating costs as disease progression required greater support:

*“I keep her money saved because I do not know how long this is going to last. I need money because if I have to care for her more days and I need to work, I need to have more than 15 hours [of support].”*

#### Transportation and mobility barriers

Transportation and mobility barriers extended beyond simple distance to include unreliable personal vehicles, limited availability of family members to provide rides, and physical or health limitations affecting both patients and caregivers. One caregiver shared:

*“I do not have transportation. I have to go by taxi. I have to take him everywhere.”*

Living without a personal vehicle further complicated both access to care and routine activities of daily living:

*“The other problem we have is that since I’m on foot, we live alone, it’s very difficult for me to take him to get his hair cut.”*

Those dependent on family assistance or those with physical mobility limitations faced further constraints:

*“Before, in terms of her mobility, I had to have my brother take me so I could get her out. I can move her, but getting her out of the car, putting her in the car was difficult.”*

Behavioral symptoms also complicated transportation, as patients sometimes resisted leaving home:

*“Sometimes he does not want to go out, does not want to get dressed. They start saying ‘because I’m here, let us go.’ It’s the condition itself.”*

### Communication and coordination failures

Communication and coordination failures were widely reported, including outdated paper-based referral processes that required in-person retrieval, lack of electronic health record (EHR) integration between providers, and multiple appointments needed to complete a single referral. One caregiver described the burden of navigating complex referral systems:

*“I left Reforma (Medicaid) because Reforma was a nightmare, because you have to chase after a referral. With the hospital, it’s so uncomfortable to park, and you have to go up to the eighth floor, and you waste time waiting for those papers to arrive.”*

Without integrated EHRs, most caregivers reported assuming primary responsibility for transmitting medical information between providers:

*“As soon as the neurologist gives us the appointment, we leave the appointment and bring all the documents, everything, to the primary care doctor so that he is aware of what is happening to her.”*

Caregivers described reconstructing the clinical history at every visit:

*“I have to explain everything to them. Each visit, I give them a history. The doctor knows, but with anything that comes up, I have to explain it.”*

This burden intensified during acute events:

*“When she was in the hospital, I’m the one who collects the papers and brings them to the primary doctor—‘Look, this happened, this, and that.’”*

### Disaster-related service disruptions

Disaster-related service disruptions included the permanent loss of health care providers and services following Hurricane María, as well as subsequent interruptions during the COVID-19 pandemic. Caregivers described the compounding and enduring impact of these disruptions on continuity of care. One caregiver whose family lost multiple providers after Hurricane María recounted:

*“Yes, we had to change because many left. Then I had to look for new specialist doctors—a new neurologist, another laboratory, a different endocrinologist. I had to leave the doctors she had because they were from that area, and access was difficult.”*

While telehealth expanded during the pandemic as an alternative to in-person visits, caregivers emphasized its limitations for dementia care. As one caregiver remarked:

*“It’s not the same—it’s not the same as in person.”*

Another explained the core clinical limitation:

*“With telemedicine, the only thing I did not like was that they could not see you—meaning they could not examine the patient,”*

Underscoring the need for in-person evaluations to assess changes in cognition, behavior, and physical function.

## Discussion

This study identified pervasive and interrelated barriers to health care access for people living with AD/ADRD in Puerto Rico. Across diverse geographic and socioeconomic contexts, caregivers described navigating a health system marked by extended wait times, limited specialist availability, and the near absence of formal systems for care coordination, leaving families themselves to serve as the primary coordinators of care. Process-level challenges such as burdensome administrative requirements and inefficient scheduling systems were especially consequential, leaving families exhausted and often forced to delay or forgo needed care. These findings reveal not only structural weaknesses in the territorial health system but also the profound human toll of chronic underinvestment.

Within Andersen’s (1995) behavioral model, these results underscore the distinction between potential and realized access. Despite near-universal insurance coverage, families frequently struggled to translate potential access into actual care utilization. Extended wait times, paper-based referral processes, and poor coordination discouraged care-seeking and undermined continuity, illustrating the feedback loops Andersen described, where negative experiences reinforce avoidance, delayed treatment, and ultimately greater burden for both families and the health system. These cycles are particularly consequential for AD/ADRD populations, for whom timely diagnosis and ongoing management are critical to slowing disease progression and maintaining caregiver stability (24). The dominance of process-level barriers suggests that policy intervention should focus on improving the organizational factors that determine whether services can be accessed effectively and efficiently. Extended wait times are particularly consequential in this population: for individuals with AD/ADRD, prolonged waits in crowded settings provoke agitation, distress, and behavioral deter future care-seeking, generating reinforcing cycles of avoidance and delayed treatment (25).

These findings extend prior research on access barriers among Medicare and dual-eligible beneficiaries on the U.S. mainland, which has generally emphasized cost-sharing and plan-switching as primary drivers of unmet need, and with prior evidence that Medicare Advantage enrollees in Puerto Rico receive systematically lower quality of care and less favorable plan financing than their mainland counterparts (8, 26). In contrast, our Medicare Advantage-dominated sample (94%) demonstrates that even comprehensive, low-cost-sharing coverage does not resolve access barriers when the underlying provider network, care coordination infrastructure, and LTSS benefit design are themselves structurally deficient. This suggests that insurance-focused policy levers, while necessary, are insufficient without complementary investment in delivery-system infrastructure, a dynamic that may be less visible in mainland studies, where LTSS coverage, though frequently inadequate, at least exists as a Medicaid benefit category. The near-universality of the barriers described here, in a population that is nearly fully insured, is itself a novel finding: it indicates that Puerto Rico’s access problem is a delivery-system and structural-financing problem rather than a coverage problem, with correspondingly different policy implications than those typically emphasized in mainland Medicare Advantage research.

The consistent absence of social needs screening across providers similarly compounds this dynamic, leaving families to identify and navigate community resources on their own. This gap has direct implications for quality of care: routine social needs screening is increasingly recognized as a core component of comprehensive dementia care management, reflected in emerging federal models such as CMS’s GUIDE Model (27), which requires participating practices to assess and address unmet health-related social needs as part of structured care planning. The near-total absence of such screening in our sample suggests that current dementia care delivery in Puerto Rico falls short of recommended standards for comprehensive care management, regardless of any single provider’s practice. Embedding brief, structured social needs screening into routine dementia care visits, paired with a defined referral pathway to community resources, represents a low-cost, near-term opportunity to improve realized access without requiring the broader structural reforms described below.

At the same time, these process failures are embedded within deeper structural inequities that constrain the island’s overall health system capacity. Critically, the core problem is not that LTSS is difficult to access but that it is architecturally absent: families were not navigating a complex system to find long-term support; they were managing its near-complete nonexistence. Chronic underinvestment, a historically fragmented financing structure, and the absence of LTSS coverage in Puerto Rico’s Medicaid program curtail essential care options for older adults and their caregivers. Although 63% of participants were dually eligible for Medicare and Medicaid, they remain categorically excluded from the LTSS benefits offered to comparable beneficiaries in the U.S. mainland (9), an inequity that exacerbates caregiver burden and accelerates functional decline for AD/ADRD patients. These domains are not independent: structural underfunding limits the organizational capacity needed for effective coordination, thereby enabling resource deficits produced by coverage gaps, and process-level failures erode families’ willingness to seek care, generating the avoidance cycles that Andersen’s model describes. Reforms targeting any single domain in isolation are therefore likely to be partially offset by failures in the others. The repercussions of outmigration following Hurricane María further compound these inequities: with clinician shortages and family support networks depleted, families face diminished access to both formal and informal care (1), forcing some to seek services off-island—Medicare beneficiary outmigration from Puerto Rico rose from 1.4 to 3.4% in the years following the hurricane, with beneficiaries diagnosed with AD/ADRD disproportionately represented among those who left, a pattern itself reflecting a failure of realized access to specialized care within the territory (28). These structural pressures compound documented psychological distress among Puerto Rico residents following Hurricane María (29), underscoring the toll of disaster-related disruption on both patients and caregivers.

These structural constraints reveal how federal policies reinforce under-resourced care systems in U.S. territories and require comprehensive reforms extending beyond health care system interventions alone (30–33). These caregiver- and patient-reported barriers converge with concurrent findings from a companion study of primary care clinicians and clinic leaders in Puerto Rico, who similarly describe chronic workforce shortages, reimbursement inadequacy, and infrastructure disruption as the central constraints on their ability to sustain continuity of care, and who identify individual clinician burden, rather than institutional support, as the primary buffer preventing system failure (13). The convergence of these independently collected caregiver- and provider-level accounts strengthens confidence that the barriers identified here reflect systemic conditions rather than isolated experiences.

Addressing these challenges requires a coordinated, multi-tiered response that targets organizational, resource, and structural levels, each corresponding to Andersen’s policy-mutable factors (19). Organizational reforms such as open-access scheduling, shown in other settings to reduce wait times by as much as 30% while decreasing no-show rates (34), shared electronic health records, and standardized care-coordination protocols could immediately improve realized access by reducing wait times and administrative bottlenecks. Strengthening enabling resources through transportation vouchers, respite care coverage, and caregiver support would enhance families’ ability to utilize available services. For rural caregivers in particular, geographic distance currently functions as a near-absolute barrier rather than a logistical inconvenience, given the concentration of specialists in metropolitan areas and the absence of publicly funded transportation support; addressing this requires targeted investment beyond generic transportation vouchers. Over the longer term, structural reforms are essential to achieve equity: establishing LTSS benefits under Puerto Rico’s Medicaid program, ensuring Medicare Advantage payment parity, and investing in disaster-resilient infrastructure to stabilize provider networks and protect access during future crises. However, these reforms face well-documented political and legislative obstacles. Federal parity for U.S. territories has historically been resisted on fiscal grounds, and proposals to expand Medicaid coverage in Puerto Rico have stalled repeatedly despite evidence of substantial unmet need (35). In the near term, administrative pathways such as expanding the scope of existing demonstration waivers, leveraging the GUIDE Model, or pursuing targeted supplemental funding for LTSS pilot programs may offer more immediately achievable entry points while longer-term structural reforms are pursued. Advocates and researchers may strengthen the case for federal action by building a robust evidence base, including cost-effectiveness analyses, that frames Medicaid parity as a federal fiscal interest rather than solely a territorial equity concern.

The dominance of process-level barriers in caregiver accounts warrants interpretive consideration. It is possible that structural barriers, precisely because they are chronic, longstanding, and normalized within Puerto Rico’s health care context, are less likely to be articulated as barriers by caregivers who have never experienced an alternative. Families who have never had access to Medicaid LTSS may not describe its absence as a barrier in the same way they would describe a four-hour wait in a clinic waiting room, because the former is invisible as an experience, whereas the latter is acutely felt. This dynamic suggests that qualitative accounts may systematically underrepresent structural barriers relative to their actual policy significance. Future research employing comparative designs across jurisdictions with and without LTSS coverage would help disentangle the relative contribution of structural and process-level factors to realized access outcomes.

### Limitations

Although the study provides a rich account of dementia care experiences in Puerto Rico, several methodological and contextual limitations must be considered. First, because participants were recruited through partner organizations and Alzheimer’s networks, the sample may overrepresent families with higher levels of engagement, awareness, or resources and may underestimate the severity of barriers among those least connected to the health care system Second, the unique forces affecting Puerto Rico’s health care system may limit generalizability, although the mechanisms affecting patient experiences are conceptually transferable to under-resourced areas of the U.S. mainland. Third, although patients participated directly in interviews as members of each dyad to the extent their cognitive status permitted, caregivers served as the primary informants and often assisted patients in responding, so the findings predominantly reflect caregiver-reported experiences and may not fully capture first-person perspectives of individuals living with AD/ADRD, particularly those with more advanced disease. Fourth, recall bias is a consideration given that some participants described experiences spanning several years, including Hurricane Maria, which occurred 6 years prior to data collection. Also, social desirability bias may have influenced responses, as caregivers may have been reluctant to speak negatively about providers. Finally, although transcripts were analyzed in their original Spanish, quotes were translated for publication; the original Spanish-language quotes are provided in Appendix 1 to allow readers to assess potential shifts in tone or emphasis.

### Policy and clinical implications

- Health systems serving older adults with dementia should implement routine social needs screening (transportation, food security, caregiver burden) and connect families to community resources.
- Policymakers should prioritize open-access scheduling models, integrated electronic health records, and standardized care coordination protocols to reduce wait times and administrative burden.
- Federal policy reforms are essential: establishing Medicaid long-term services and supports coverage in Puerto Rico, ensuring Medicare Advantage payment parity with mainland states, and investing in disaster-resilient health infrastructure.

## Data Availability

The data supporting the findings of this study contain sensitive, potentially identifying information about individuals living with AD/ADRD and their family caregivers, collected under IRB-approved protocols that assured participants of confidentiality; the underlying interview transcripts are therefore not publicly available. De-identified excerpts and coded data are available from the corresponding author upon reasonable request, subject to approval by the Institutional Review Boards of the University of Puerto Rico, Medical Sciences Campus, and RAND, and execution of a data use agreement to protect participant confidentiality. Requests to access the datasets should be directed to Justin Timbie, jtimbie@rand.org.
