# Supplementary material for: Barriers to health care access in a federally under-resourced system: experiences of Alzheimer’s disease and related dementias patients and caregivers in Puerto Rico
Source: Front Public Health. 2026 Oct 8;14:1923342. doi: 10.3389/fpubh.2026.1923342 (PMC13687668; doi:10.3389/fpubh.2026.1923342)
Supplement: Supplementary file 1 [file Supplementary_file_1.pdf]

Appendix 1. Original Spanish-Language Quotes

This appendix presents the original Spanish-language text from participant interviews, along with English translations, as published in the main text, organized by thematic section. All Spanish text is verbatim from the Dedoose-coded excerpt database.

Structural Barriers

LTSS Coverage Gaps

Quote 1

English (as published):

> *"She is 24/7. She needs someone. She doesn't remember anything. She doesn't feed herself. She doesn't go to the bathroom by herself. She's already wearing diapers. She doesn't recognize absolutely anyone."*

Original Spanish (verbatim):

> *"Ella es 24-7. Ella necesita alguien. Así que ella no se acuerda de nada. Ella no se alimenta por sí sola. Ella no va al baño por sí sola. Ella ya está usando pañales. Ella no reconoce. Como dije, ella está en la última etapa. Así que ella no reconoce absolutamente a nadie."*

Quote 2

English (as published):

> *"It would be ideal to have these services available, either through your insurance plan or government assistance, but there's nothing."*

Original Spanish (verbatim):

> *"Sería ideal tener estos servicios disponibles, ya sea que uno lo pueda pagar con el plan médico o ayudas del gobierno, pero no, no hay nada."*

Quote 3

English (as published):

> *"At the stage he's in now, that's what he needs. Therapy and physical maintenance help... It's very important that he delays becoming bedridden."*

Original Spanish (verbatim):

> *"Para mi papá entiendo que es muy importante que sería muy bueno tener esta oportunidad de que él tuviera esos servicios de terapias cognitivas y de mantenimiento físico. Para los caminos por los que él esta es bien importante el que se atrasa en llegar a una cama, si no se atrasa cualquier paciente no nada más que una persona con el diagnóstico."*

Specialist Access and Provider Network Issues

Quote 4

English (as published):

> *"Specialists are difficult to access. With the neurologist—goodness—finding a neurologist these days is an uphill battle. There is no special treatment [for people living with Alzheimer's] for getting an appointment."*

Original Spanish (verbatim):

> *"Es que los especialistas están difíciles al acceso. Con el neurólogo, muchacha. Seguir un neurólogo en estos tiempos es cuesta arriba. No hay un trato especial para conseguir la cita."*

Quote 5

English (as published):

> *"There are neurologists I have called who are not taking new patients. And it has been very difficult. The same with psychiatrists."*

Original Spanish (verbatim):

> *"Hay neurólogos que he llamado que no están cogiendo pacientes nuevos. Y pues se han hecho difícil. Igual que los psiquiatras."*

Quote 6

English (as published):

> *"They removed him from the health plan we had and I switched health plans. Just to keep him. Because he treats her very well and has been with her for several years, and honestly, at her age, changing to a new doctor [is difficult]."*

Original Spanish (verbatim):

> *"Lo sacaron del plan médico que teníamos y me cambié de plan médico. Para quedarnos con el. Porque él la trata muy bien y lleva varios años con él y entonces, a la verdad, a la edad que ella tiene cambiarla con un médico nuevo."*

Quote 7

English (as published):

> *"To get a urologist it has to be one that the plan covers—and most urologists do not take insurance."*

Original Spanish (verbatim):

> *"El único problema es que para conseguir un urólogo tiene que ser lo que el plan tiene y la mayor parte de los urólogos no cogen plan. No lo quieren coger."*

Care Process Barriers

Extended Wait Times

Quote 8

English (as published):

> *"Appointments with him require waiting four hours—in the waiting room. So yes, for me to take him, I prefer to start early, in the morning."*

Original Spanish (verbatim):

> *"Las citas de él tienes que esperar 4 horas. Esperando en la sala. Entonces, sí, para yo cogerlo, que prefiero empezar temprano, en la mañana."*

Quote 9

English (as published):

> *"For example, I have an appointment at 11 and they schedule four people for 11. At 11:30 still none of the four have been seen."*

Original Spanish (verbatim):

> *"Por ejemplo, me cita para las 11 y puede citar a las cuatro personas para las 11. A las 11 y media todavía no haya visto ninguno de los cuatro."*

Quote 10

English (as published):

> *"It distresses her too, and many people, because the offices were completely full. I didn't want to expose her to that."*

Original Spanish (verbatim):

> *"No tenía que ir a la oficina a esperar mucho tiempo porque eso la desespera también, mucha gente porque las oficinas se pasaban llenas, llenas, llenas, no la iba a exponer tampoco."*

Lack of Social Needs Screening

Quote 11

English (as published):

> *"I don't think they ever ask, unless they see someone really malnourished."*

Original Spanish (verbatim):

> *"No creo que pregunten nunca dentro de, a menos que vean alguien bien desnutrido."*

Quote 12

English (as published):

> *"They only communicate with you if they see an urgent need. If there's no urgency, or you don't raise the concern yourself, they don't ask."*

Original Spanish (verbatim):

> *"Si es urgente, ellos sí. Ellos ven que en algún momento el paciente tenía un problema de hogar o con la aportación, pues entonces ellos se acercan a uno y llaman para ver cómo está. Pero si no hay esa urgencia o uno no da esa queja o no pone esa preocupación, ellos no preguntan."*

Quote 13

English (as published):

> *"They've never asked if we needed help. Everything I have and know, I found on my own."*

Original Spanish (verbatim):

> *"Por ellos jamás. Tuve que buscarlo por mi propia cuenta."*

Enabling Resource Barriers

Geographic Barriers

Quote 14

English (as published):

> *"When she had an episode (Transition Ischemic Attack) and left the hospital, obviously she couldn't be alone. I brought both of them [home with me], and I had to change all their doctors to the metropolitan area so I wouldn't have to be running around constantly."*

Original Spanish (verbatim):

> *"Ella tuvo un episodio, le dio un TIA, un Transition Ischemic Attack en el 22. Entonces cuando salió del hospital, obviamente no podía estar sola, yo me los traigo a los dos, tuve que cambiar todos sus médicos para el área metropolitana para no tener que estar dando carrera."*

Quote 15

English (as published):

> *"I searched for places where she could go to a day center. There aren't any available in the municipality of San Juan. They are available in Carolina, but they don't accept her because of her address."*

Original Spanish (verbatim):

"Busqué lugares donde pudiera estar diurnos. No los hay disponible en el municipio de San Juan. Hay disponible en Carolina. Y no la aceptan por la dirección."

Quote 16

English (as published):

"It's like the services are for the metropolitan area, but here in Humacao it's completely different."

Original Spanish (verbatim):

"Es como para el área metropolitana, pero aquí en Humacao nada que ver."

Financial Barriers

Quote 17

English (as published):

"The original medications... the plan doesn't cover them, and I can't pay for them. I have to find a way and use the generic ones... For example, the eye drops they need... but they cost $500."

Original Spanish (verbatim):

"Las originales son bien, que no las cubre el plan, y no las puedo pagar. Tengo que buscar la forma y las genéricas no son muy buenas, pero me he tenido que adaptar a ellas. Por ejemplo, ellos necesitan las gotas que se llaman con picanas, pero valen 500 dólares."

Quote 18

English (as published):

"I found another alternative place that's really cool but super expensive—almost $900 a month. Who can pay that?"

Original Spanish (verbatim):

"Busqué otro sitio alternativo, información en un centro que se llama XXX o algo así que es súper chévere pero súper caro, casi 900 dólares mensuales ¿Quién puede pagar eso?"

Quote 19

English (as published):

"I keep her money saved because I don't know how long this is going to last. I need money because if I have to care for her more days and I need to work, I need to have more than 15 hours [of support]."

Original Spanish (verbatim):

"Los chavitos de ella se los cuido para que duren porque yo no sé hasta cuánto va a ser esto. Necesito que tengo chavos porque si tengo que cuidarla más días y necesito trabajar o... ya vamos al médico, tengo que tener más de 15 horas. Y ese osea el cuidado."

Transportation and Mobility Barriers

Quote 20

English (as published):

"I don't have transportation. I have to go by taxi. I have to take him everywhere."

Original Spanish (verbatim):

> *"No tengo transportación, tengo que irme en taxis, tengo que llevarlo entre todas esas cosas."*

Quote 21

English (as published):

> *"The other problem we have is that since I'm on foot, we live alone, it's very difficult for me to take him to get his hair cut."*

Original Spanish (verbatim):

> *"El otro problema que tenemos es que como estoy a pie, vivimos solos, pues se me hace bien difícil para llevarlo a recortar, que le recorten los cabellos de la cabeza, los cabellos de los oídos, los cabellos de la nariz."*

Quote 22

English (as published):

> *"Before, in terms of her mobility, I had to have my brother take me so I could get her out. I can move her, but getting her out of the car, putting her in the car was difficult."*

Original Spanish (verbatim):

> *"Antes en termino por la movilidad de ella ahí ya sabes tenía que llevarla mi hermano me tenía que llevar para poderla sacar sabes yo la puedo mover tú sabes pero sacarla del carro, meterla en el carro era difícil."*

Quote 23

English (as published):

"Sometimes he doesn't want to go out, doesn't want to get dressed. They start saying 'because I'm here, let's go.' It's the condition itself."

Original Spanish (verbatim):

"La condición misma, es la condición misma porque a veces no quiere salir, no se quiere vestir. Ellos empiezan 'porque estoy aquí, ya vámonos'. Es la condición misma."

Communication & Coordination Failures

Quote 24

English (as published):

"I left Reforma (Medicaid) because Reforma was a nightmare, because you have to chase after a referral. With the hospital, it's so uncomfortable to park, and you have to go up to the eighth floor, and you waste time waiting for those papers to arrive. "

Original Spanish (verbatim):

"Yo salí de Reforma porque Reforma era una pesadilla, porque tienes que estar detrás de un referido, pedir referido y aparte de que pidiera el referido, entonces la doctora tenía un día para el referido, tú ibas a buscar el referido y ah, no está hecho, tienes que ir a la otra semana a buscar el referido."

Quote 25

English (as published):

"As soon as the neurologist gives us the appointment, we leave the appointment and bring all the documents, everything, to the primary care doctor so that he is aware of what is happening to her."

Original Spanish (verbatim):

> *"Tan pronto la neuróloga nos da la cita, salimos de la cita, le llevamos todos los documentos, todo al médico de cabecera para que esté al tanto de lo que le está pasando."*

Quote 26

English (as published):

> *"I have to explain everything to them. Each visit, I give them a history. The doctor knows, but with anything that comes up, I have to explain it."*

Original Spanish (verbatim):

> *"Si, yo le tengo que explicar todo... Si. por lo menos el neurólogo no, pero sí el generalista... o sea, él sabe, pero cualquier cosa pues le tengo que explicar."*

Quote 27

English (as published):

> *"When she was in the hospital, I'm the one who collects the papers and brings them to the primary doctor — 'Look, this happened, this, and that.'"*

Original Spanish (verbatim):

> *"Cuando ella estuvo en el hospital, no fue con el generalista. No fue con su médico de cabecera, porque esos médicos ahora no pueden hospitalizar. Cuando yo vine a la sala de emergencia, la hospitalizaron, yo soy la que cojo los papeles y se los llevó a él, 'Mire me pasó esto, esto y esto'."*

Disaster-related service disruption

Quote 28

English (as published):

"Yes, we had to change because many left. Then I had to look for new specialist doctors—a new neurologist, another laboratory, a different endocrinologist. I had to leave the doctors she had because they were from that area, and access was difficult."

Original Spanish (verbatim):

"Si, hubo que cambiar porque muchos se fueron, entonces tuve que buscar médicos especialistas nuevos. Tuve que buscar un neurólogo nuevo, otro laboratorio nuevo, el endocrinólogo es diferente, tuve que dejar los médicos que ella tenía porque eran de esa área, y fue difícil el acceso.

Quote 29

English (as published):

"It's not the same—it's not the same as in person."

Original Spanish (verbatim):

"No es lo mismo, no es lo mismo físicamente."

Quote 30

English (as published):

"With telemedicine, the only thing I didn't like was that they couldn't see you—meaning they couldn't examine the patient."

Original Spanish (verbatim):

"Las de telemedicina, lo único que no me gustaba era que no te veía, o sea que no examinaban al paciente."
