# Supplementary material for: Barriers to health care access in a federally under-resourced system: experiences of Alzheimer’s disease and related dementias patients and caregivers in Puerto Rico
Source: Front Public Health. 2026 Oct 8;14:1923342. doi: 10.3389/fpubh.2026.1923342 (PMC13687668; doi:10.3389/fpubh.2026.1923342)
Supplement: Supplementary file 2 [file Supplementary_file_2.pdf]

Appendix 2. Coding and Analysis Process

We analyzed 1,162 coded excerpts in Dedoose using a systematic qualitative approach that documented all coding decisions and the development of themes. The process involved familiarization with Spanish transcripts through repeated reading; line-by-line coding using both Andersen's framework-based and emergent codes; classification of barriers as affecting potential or realized access; assessment of mutability following Andersen (12); inductive cycles to identify recurrent terminology; constant comparative analysis to confirm pattern consistency; cultural-linguistic validation by native Spanish-speaking team members; inter-coder reliability testing through independent coding; and consensus meetings to resolve discrepancies.

Barrier-Specific Linguistic Patterns

Through iterative manual coding, we identified consistent Spanish-language patterns that indicated health care barriers, categorized according to their impact on potential versus realized access:

Potential Access Barriers (System Availability)

- **Absence Terms:** "no hay" (there isn't/aren't), "no existe" (doesn't exist), "cerrado" (closed)
- **Limitation Language:** "limitado" (limited), "restringido" (restricted), "falta" (lack/shortage)
- **Coverage Gaps:** "no cubre" (doesn't cover), "sin beneficio" (without benefit)

Realized Access Barriers (Utilization Challenges)

- **Process Difficulties:** "difícil" (difficult), "complicado" (complicated), "imposible" (impossible)
- **Wait/Delay Terms:** "esperar" (to wait), "tardarse" (to take time/delay), "demora" (delay)

- **Navigation Problems:** “no puede” (can’t), “problema” (problem), “lento” (slow)

Enabling Resource Barriers

- **Financial Constraints:** “caro” (expensive), “dinero” (money), “pagar” (to pay)
- **Distance/Access Terms:** “lejos” (far), “transporte” (transportation), “llegar” (to arrive/reach)

Communication & Coordination Failures

- **Referral/Documentation Terms:** “referido” (referral), “papeles” (papers/documents), “expediente” (medical record)
- **Repetition Language:** “explicar de nuevo” (explain again), “repetir” (to repeat), “cada vez” (every time)
- **Communication Gaps:** “no se comunican” (they don’t communicate), “no saben” (they don’t know)

Post-Disaster Feedback Effects

- **Temporal References:** “después de María” (after María), “perdimos servicios” (we lost services)
- **Provider Loss:** “se fueron” (they left), “no regresó” (didn’t return)

The analysis focused on identifying explicit barriers to health care access while preserving participant voice and cultural context throughout all phases of analysis, with particular attention to distinguishing barriers that represent policy-mutable factors from those representing immutable characteristics.
